# Supplementary material for: Structural insights into the Ca2+-dependent gating of the human mitochondrial calcium uniporter
Source: eLife. 2020 Aug 7;9:e60513. doi: 10.7554/eLife.60513 (PMC7442490; doi:10.7554/eLife.60513)
Supplement: Figure 1—source data 1. [file elife-60513-fig1-data1.doc]

|  | **Ca^2+^-bound** | **Ca^2+^-free** | | |
| --- | --- | --- | --- | --- |
|  |  | **Blocking** | **Bridging** | **Competing** |
|  | **EMDB: EMD-22215**  **PDB: 6XJV** | **EMDB: EMD-22216**  **PDB: 6XJX** | **EMDB: EMD-22213** | **EMDB: EMD-22214** |
| **Data collection and processing** |  |  |  |  |
| Magnification | 105k | 105k | 105k | 105k |
| Voltage (kV) | 300 | 300 | 300 | 300 |
| Electron exposure (e–/Å^2^) | 62 | 62 | 62 | 62 |
| Defocus range (μm) | -0.9 ~ -2.2 | -0.9 ~ -2.2 | -0.9 ~ -2.2 | -0.9 ~ -2.2 |
| Pixel size (Å) | 0.833 | 0.833 | 0.833 | 0.833 |
| Symmetry imposed | C1 | C1 | C1 | C1 |
| Initial particle images (no.) | 694,082 | 2,070,698 | 2,070,698 | 2,070,698 |
| Final particle images (no.) | 19,924 | 44,681 | 44634 | 12621 |
| Map resolution (Å) | 4.17 | 4.60 | 4.50 | 7.06 |
| FSC threshold | 0.143 | 0.143 | 0.143 | 0.143 |
| Map resolution range (Å) | 299.88 ~ 4.17 | 266.56 ~ 4.60 | 359.86 ~ 4.50 | 359.86 ~ 7.06 |
|  |  |  |  |  |
| **Refinement** |  |  |  |  |
| Model resolution (Å) | 4.17 | 4.60 |  |  |
| FSC threshold | 0.143 | 0.143 |  |  |
| Model resolution range (Å) | 299.88 ~ 4.17 | 266.56 ~ 4.60 |  |  |
| Map sharpening *B* factor (Å^2^) | -115 | -162 |  |  |
| Model composition |  |  |  |  |
| Non-hydrogen atoms | 29,921 | 12,077 |  |  |
| Protein residues | 3,649 | 1,462 |  |  |
| Ligands | 0 | 0 |  |  |
| *B* factors (Å^2^) | 93.61 | 146.34 |  |  |
| Protein | 93.61 | 146.34 |  |  |
| Ligand | 93.61 | 146.34 |  |  |
| R.m.s. deviations |  |  |  |  |
| Bond lengths (Å) | 0.006 | 0.009 |  |  |
| Bond angles (°) | 0.980 | 1.175 |  |  |
| Validation |  |  |  |  |
| MolProbity score | 1.57 | 1.86 |  |  |
| Clashscore | 4.40 | 8.05 |  |  |
| Poor rotamers (%) | 0.27 | 0.39 |  |  |
| Ramachandran plot |  |  |  |  |
| Favored (%) | 94.87 | 93.61 |  |  |
| Allowed (%) | 4.97 | 6.39 |  |  |
| Disallowed (%) | 0.17 | 0 |  |  |

**Figure 1—source data 1. Cryo-EM data collection, refinement and validation statistics**
